# American Mock World Health Organization: An Innovative Model for Student Engagement in Global Health Policy

**DOI:** 10.9745/GHSP-D-16-00138

**Published:** 2017-03-24

**Authors:** Mia Lei, Neha Acharya, Edith Kwok Man Lee, Emma Catherine Holcomb, Veronica Kapoor

**Affiliations:** a University of North Carolina, Chapel Hill, NC, USA.; b Rollins School of Public Health, Emory University, Atlanta, GA, USA.; c Fielding School of Public Health, The University of California, Los Angeles, Los Angeles, CA, USA.

## Abstract

The American Mock World Health Organization (AMWHO) is a model for experiential-based learning and student engagement in global health diplomacy. AMWHO was established in 2014 at the University of North Carolina at Chapel Hill with a mission to engage students in health policy by providing a simulation of the World Health Assembly (WHA), the policy-forming body of the World Health Organization that sets norms and transforms the global health agenda. AMWHO conferences are designed to allow students to take their knowledge of global health beyond the classroom and practice their skills in diplomacy by assuming the role of WHA delegates throughout a 3-day weekend. Through the process of developing resolutions like those formed in the WHA, students have the unique opportunity to understand the complexities behind the conflict and compromise that ensues through the lens of a stakeholder. This article describes the structure of the first 2 AMWHO international conferences, analyzes survey results from attendees, and discusses the expansion of the organization into a multi-campus national network. The AMWHO 2014 and 2015 post-conference survey results found that 98% and 90% of participants considered the conference "good" or "better," respectively, and survey responses showed that participants considered the conference "influential" in their careers and indicated that it "allowed a paradigm shift not possible in class."

## Abstract (precis)

The American Mock World Health Organization (AMWHO) provides a platform for students to apply their knowledge of global health policy through simulations of the World Health Assembly (WHA). This model engages and empowers future global leaders in health policy while sharpening their skills in diplomacy, public speaking, and conflict resolution. The major theme for the AMWHO 2015 was Universal Health Coverage, reflecting what the WHA had discussed in preceding months.

## BACKGROUND

In January 2014, founder and author Neha Acharya created the American Mock World Health Organization (AMWHO) at the University of North Carolina at Chapel Hill, based on the Ontario Model World Health Organization (OMWHO) in Toronto, Canada, an organization holding conferences that simulate the annual World Health Assembly (WHA) held in Geneva, Switzerland. Its sister structure, Model United Nations, replicates the United Nations General Assembly, and is widely popular in both high schools and universities throughout the world. Within the United States, few students have similar opportunities to sharpen skills and explore a future in health policy. Mock World Health Organization conferences fill that gap by engaging students in health diplomacy by replicating the WHA debating a particular, complex global health issue.

While attending OMWHO, author Neha Acharya observed that diplomacy was key to realizing the goals of global health, a concept and skill not prevalent in global health policy curricula. As Ilona Kickbusch and coauthors note, it is necessary for public health professionals to have training in diplomacy and for diplomats to have training in public health to build necessary capacity in health diplomacy.^1^ Yet while Lee and Smith agree that "negotiation of health-related agreements can benefit from the skills and experience of diplomats," they argue that "in the past, the ‘toolbox’ of the health policy maker has not included expertise in international negotiation."^2^ With an increasingly integrated and globalizing world, future global health leaders are faced with multifaceted issues that require a skill set in negotiating and forming policies with other global health players. WHO's significant challenges in effectively cooperating with key stakeholders make diplomacy training a practical tool that can better prepare aspiring public health professionals to listen, negotiate, and develop solutions.^3^

Future global health leaders require a skill set in negotiating and forming policies with other global health players.

Since the founding 2014 AMWHO conference, 2 international AMWHO conferences have been held. In this article, we describe the structure and procedures of the AMWHO conference and roles that students play while illustrating the content of the debate with a case study from the Americas region during the AMWHO 2015 conference. We also present findings from online surveys of AMWHO 2014 and 2015 participants to inform successes and opportunities for growth.

## STRUCTURE OF AN AMWHO CONFERENCE

### Conference Roles

During each 3-day mock WHO conference, delegates assume the role of either a WHO Member State Ambassador, an NGO representative, or a media correspondent. Most delegates represent WHO member states as they debate, strategize, and form resolutions. A handful of delegates represent NGOs and the media. Reflective of the role of NGOs in global health governance, resolutions proposed by WHO Member State delegates can only pass with approval from a majority of the NGO delegates. Representing the role of the media in global health governance, media correspondents move between committees to report on the proceedings of debate, often influencing the direction of discussion.

At the American Mock World Health Organization, student delegates assume the role of either a WHO Member State Ambassador, an NGO representative, or a media correspondent.

Dais members are students from the host university who are trained in parliamentary procedures to ensure a realistic WHA simulation. Three key actors sit on the dais of each committee and facilitate debate during committee sessions:

- Chair: facilitates debate and ensures that procedural matters follow Robert's Rules of Order
- Vice Chair: assists delegates with the processes of drafting and approving resolutions
- Rapporteur: keeps committee order by maintaining the speakers' list, calling roll, tracking all resolutions and votes, and facilitating communiques between delegates

### Conference Schedule

Participants spend the first 2 days of the conference in *committee sessions*, where they debate how to best address the chosen theme, negotiate draft working resolutions, and gather support for their proposed plan of action. They spend the third day in *plenary*, where all regional committees convene to amend and finalize draft resolutions (Table 1). Subject matter experts provide insight into the theme at scheduled presentations throughout the conference. The conference ends with closing ceremonies, where awards are announced for Best Delegate and Best Position Paper from each region.

**TABLE 1.** Sample American Mock World Health Organization Conference Schedule

Day and Time	Program
**Friday**	
1:00–1:45 pm	Delegate Training
1:45–3:00 pm	Opening Ceremonies
3:00–6:00 pm	Committee 1
6:00–7:00 pm	Speaker 1
7:00–9:00 pm	Delegate Dinner
**Saturday**	
8:30–9:00 am	Breakfast
9:00 am–12:00 pm	Committee 2
12:00–2:00 pm	Lunch & Learn
2:00–4:00 pm	Committee 3
4:00–5:00 pm	Speaker 2
5:00–7:00 pm	Committee 4
7:30–9:00 pm	Delegate Social
**Sunday**	
8:30–9:00 am	Breakfast
9:00 am–12:00 pm	Plenary 1
12:00–1:15 pm	Lunch
1:15–2:15 pm	Plenary 2
2:15–3:30 pm	Keynote Speaker
3:30–4:30 pm	Plenary 3
4:30–5:15 pm	Closing Ceremonies

### Committee Sessions

#### Set Up and Training

Committee sessions comprise the majority of the first 2 days. During committee sessions, WHO Ambassador delegates convene in regional blocs based on the WHO regions: Africa (AFRO), the Americas (AMRO), Europe (EURO), Eastern Mediterranean (EMRO), South-East Asia (SEARO), and Western Pacific (WPRO). SEARO and WPRO are combined into 1 region for the conference to allow for a substantive amount of delegate representation in the region.

**Figure:**
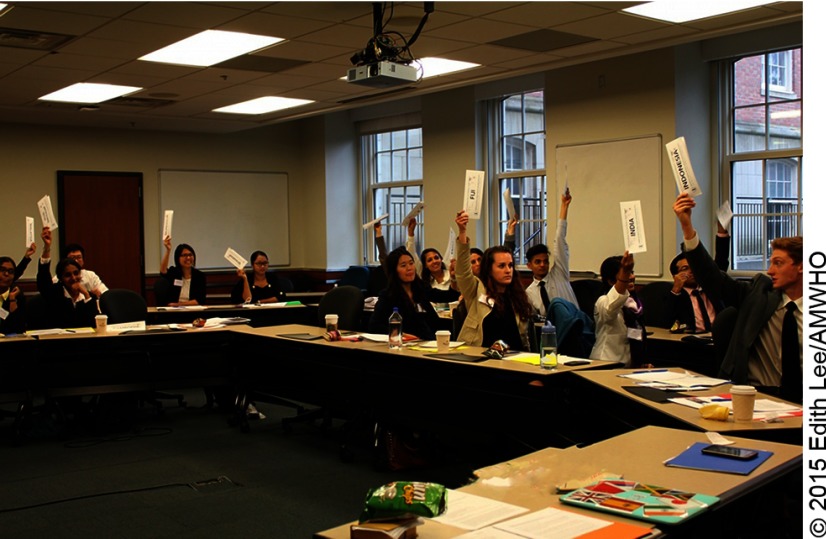
Delegates in the South-East Asia (SEARO) and Western Pacific (WPRO) committee in moderated caucus.

The students convene in regional blocs at committee sessions during the first 2 days of the conference.

On Day 1, AMWHO conferences begin with a delegate training session to familiarize the students with parliamentary procedure and the overall conference. The first committee session follows shortly thereafter. Within this session, delegates present ideas on setting the resolution agenda to a narrower, region-specific subtheme of the main conference theme. All subsequent resolutions are written on the agenda topic, which is set by majority vote.

#### Subject Matter Experts

After delegate training on Day 1, an opening ceremony features a subject matter expert who introduces the delegates to the conference theme. This speaker provides a framework and context for delegates to better understand and write their position paper on the topic of debate. Throughout the remaining days of the conference, 2 to 3 subject matter experts speak to provide insight from their fieldwork, research, and other experiences. See Table 2 for a list of speakers from the AMWHO 2014 and 2015 conferences.

**TABLE 2.** Speakers at the American Mock World Health Organization 2014 and 2015 Conferences

Year	Day 1	Day 2	Day 3
2014	Steven Wayling: former technical officer with the Global Programme on AIDS in the WHO/EURO office in Copenhagen	Heather Davis: worked with PEPFAR and CARE in Ethiopia and the DRC	Mory Pagel: worked with SIT Study Abroad on field-based research within the WHO in Geneva, Switzerland
2015	Dr. Audrey R. Chapman: professor in the Division of Public Health Law and Bioethics, and the first Healey Endowed Chair in Medical Ethics and Humanities at the University of Connecticut Health Center	Dr. Timothy G. Maestro: Director of Global Health, Population and Nutrition at FHI 360	U.S. Ambassador Jimmy Kolker: Assistant Secretary for Global Affairs at the U.S. Department of Health and Human Services and the U.S. representative to the WHO under President Barack Obama

Abbreviations: DRC, Democratic Republic of the Congo; PEPFAR, The United States President's Emergency Plan for AIDS Relief; SIT, School for International Training; WHO/EURO, World Health Organization Regional Office for Europe.

#### Debate Procedures

On Day 2, committee sessions continue, and delegates engage in discourse on the best solutions to the committee subtheme. Delegates address the entire committee in *moderated caucuses* and obtain consensus on their proposed plan of action. Delegates later enter into an *unmoderated caucus* for informal discussions that move toward drafting resolutions.

#### Resolutions

Each region can form up to 2 resolutions. Between 1 and 3 *Sponsors* write the resolution and are responsible for seeing its passage. *Signatories* support the spirit of the resolution but may not fully agree with the entirety of the clauses. By the last committee session of Day 2, fully formed draft resolutions are submitted to the dais. At this time, delegates may amend each other's resolutions by adding or removing clauses, altering language, or adding specificity through sub-clauses. At the end of the day, each regional committee votes on all amended draft resolutions. If passed, the draft resolution goes to plenary on the last day for consideration by all member state representatives.

#### Plenary

Delegates from all regional blocs convene in plenary on the final day of the conference. Sponsors present an overview of each resolution, and the conference as a whole amends and votes on the regional resolutions. Those that pass plenary are sent to the World Health Organization in Geneva for review.

Student delegates from all regional blocs convene in plenary on the final day of the conference.

## PUBLICITY AND OUTREACH

AMWHO recruited both undergraduate and graduate students through social media marketing, through the American Public Health Association, and emails to individual professors and to university listservs. Table 3 outlines the universities represented at both international conferences, as well as universities hosting regional or local conferences. AMWHO also received sponsorship from local and global health organizations.

**TABLE 3.** University Involvement in AMWHO Activities

University	Represented at AMWHO 2014 and/or 2015	Chapter Organization	Regional Conference Host
University of North Carolina at Chapel Hill	✓	✓	✓
Emory University	✓	✓	✓
Virginia Tech	✓	✓	✓
University of Kentucky	✓	✓	✓
University of South Carolina	✓	✓	
Mercer University	✓	✓	
University of Georgia	✓	✓	✓
University of Denver	✓	✓	
Cornell University	✓	✓	✓
University of Washington	✓	✓	✓
Johns Hopkins Bloomberg School of Public Health	✓	✓	
Case Western Reserve University		✓	✓
…and 21 more universities	✓		

Abbreviation: AMWHO, American Mock World Health Organization.

## CONTENT OF DEBATE

The AMWHO 2015 conference selected the theme of "Universal Health Coverage" to in alignment with a topic discussed by the WHA in the months preceding the conference. Table 4 describes the content of discussions and debates that occurred in each region during this conference, and the Box provides more details about the debate that emerged specifically from the AMRO region. The experience concluded at plenary, where AMRO's resolution was passed with the addition of a few amendments. See the Appendix for the AMRO resolution that resulted from the plenary.

**TABLE 4.** Determination of Resolution Topics at the American Mock World Health Organization 2015 Conference, by Region/Committee

Region	Proposed Subthemes	Selected Subtheme (Agenda)	Resolution Title	Resolution Topics
African Region (AFRO)	Sustainable health systems Diverse funding sources for health care Teaching, managing, and organizing community health workers Health education reform Novel measurement and evaluation methods for health interventions Emphasis on incorporating social determinants of health in health policy aimed at universal health coverage	Creating sustainable health systems in all African nations and achieving universal health coverage by holistically innovating health care infrastructure to meet nation-specific needs	The creation of sustainable health systems	Retention, education, and training of a competent health care workforce Methods to improve health education to educate different communities and demographics Strengthening relationships between nations and NGOs Creation of a novel framework to create and develop sustainable health systems Addressing the health care needs of refugees
Americas Region (AMRO)	Primary care accessibility Definition of essential medicines Health outcomes measurement Health financing Health workforce and resources Vulnerable and neglected populations	Ensuring universal health coverage as defined by equitable access to health services for all, with an emphasis on access to care for vulnerable populations.	Ensuring universal health coverage as defined by equitable access to health services for all, emphasizing access to care for vulnerable populations	Health information systems integration Definition of essential medicines Financial support Service expansion and sustainable development Appropriate training of community health workers
Eastern Mediterranean Region (EMRO)	Community health worker training and curriculum development Supportive health care units during times of crisis Training primary health care doctors for both practice and retention in EMRO Designing and implementing a crisis package for universal health coverage in conflicts areas Universal policy to guarantee security and sustainability during times of natural and welfare crisis Package that focuses on data analysis and primary health care delivery to target populations, encourages the increase of sanitation and water access across EMRO, sets aside a crisis fund, and improves medical education to train and retain primary health care doctors during times of crisis	Implementing universal health coverage including building the framework for health analytics, crisis funding, primary health care funding, education of medical professionals, and security within health care infrastructures	Immediate Relief Universal Health Care Package for Times of Crisis	Provision of immediate relief resources Establishment of an EMRO crisis fund Creation of longitudinal health care approaches Developing partnerships between governments and centers of excellence
European Region (EURO)	Education of health care workforce to address quantity and specialization of health care providers Access to health care services for vulnerable populations Bolstering preventative care to increase health care efficiency Privatization of universal health care for health financing Surveillance and sustainability of health care workforce	Addressing accessibility and availability of health care workforce as a means of providing universal health coverage with a focus on outreach to vulnerable populations	Increase the number, training, quality, and equitable distribution of workforce	Restructuring of health care education Redistribution of health care workforce Financial sustainability and surveillance of novel educational programs
South-East Asian Region and Western Pacific Region (SEARO/WPRO)^a^	Quality of service Equal and affordable access Rural access Emergency services Financing universal health coverage	Financing universal health care with a focus on improving service quality and decreasing health inequality	Financial Support for SEARO/WPRO Health Initiatives	Creation of a regional financial management committee to manage and administer funds Cost-sharing for NGOs and governments Decentralized programs Monitoring and assessment of efficient fund allocation

a For the purposes of creating similar-sized committees, SEARO and WPRO, the 2 smallest regions of the WHO, were combined for all AMWHO conferences.

BOX. **Case Study of the Americas Region Committee Debate at the American Mock World Health Organization 2015 Conference**

The theme of AMWHO 2015 was Universal Health Coverage. Over the 3-day conference, most participants were grouped into regional committees, based on the WHO regional offices. Some participants assumed the roles of NGO representatives or media correspondents. This case study of the Americas Region (AMRO) committee highlights illustrative examples of the experience of participants.

**Day 1 Committee Session: Setting the Resolution Agenda**

Upon arrival, each delegate had identified the issues relevant to his/her particular country in the form of position papers. The first committee session began in a disjointed fashion when delegates were given their initial opportunity to speak as a wide variety of concerns were presented.

Under the theme of universal health coverage, the dominant topics in AMRO were primary care accessibility, definitions of essential medicine, health outcomes measurement, health financing, and health workforce and resources. Delegates formed a consensus on the following established agenda: “Ensuring universal health coverage as defined by equitable access to health services for all, with an emphasis on access to care for vulnerable populations.”

**Day 2 Committee Sessions: Negotiating Draft Resolutions**

Delegates aimed to clarify their agenda by agreeing upon key definitions. A comprehensive definition of vulnerable populations ultimately served as a pre-ambulatory clause to the resolution, which set the tone to recognize that each country has distinctive marginalized groups. Further discussion was also needed to define the intent of the phrase “equitable access to all” in the agenda; the delegates ultimately settled upon calling for child health care, primary care, and preventative care. Several countries advocated the inclusion of mental health in this definition, but it was not included.

Once these goals were more clearly defined, debate turned to topics that structurally supported the chosen agenda subtheme. Many of the dominant topics not chosen as the subtheme were later addressed as subtopics in the agenda. The committee discussed the importance of health information systems, accounting for financial hardships, and expansion of health services into underserved regions. Health information systems were proposed to monitor outcomes and thus assess the success of various universal health coverage systems. Finances were tackled with a call for collaboration between countries on international issues as well as better coordination of existing funds within countries.

Throughout debate, the delegates worked within the WHO's scope of power and avoided infringing upon any country's sovereignty. As delegates moved to unmoderated caucus to write resolution clauses, NGOs requested to modify clauses to maintain their influence but rejected any clause that would impose excessive responsibility upon them. Resolution sponsors then presented the draft resolutions for amendments and approval. Among various amendments that dealt with slight wording changes, AMRO voted to include the aforementioned clause concerning mental health, which was pivotal for some delegates whose votes were unsecured. Due to the dichotomy of wealth in the region, the committee did not pass a contentious amendment that acknowledged responsibility to donor countries.

U.S. Ambassador Jimmy Kolker, keynote speaker to the AMWHO 2015 conference, also participated during the final AMRO committee session. Ambassador Kolker briefly represented the United States and offered a few amendments, including one that added lesbian, gay, bisexual, transgender, and queer/questioning (LGBTQ) to the definition of vulnerable populations, which passed unanimously.

**Figure:**
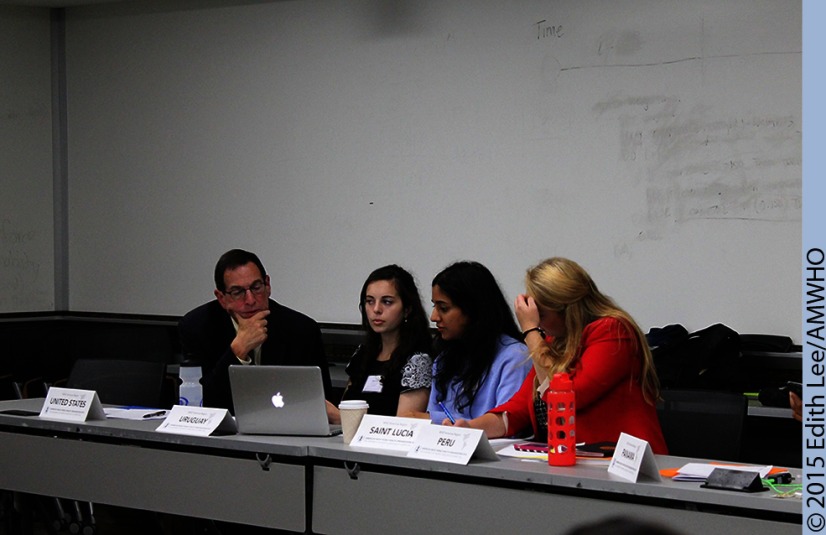
U.S. Ambassador Jimmy Kolker, Assistant Secretary for Global Affairs at the U.S. Department of Health and Human Services and the U.S. representative to the WHO, works with students on a resolution at the American Mock World Health Organization 2015 conference.

**Day 3 Plenary: Convening All Regional Committees to Amend and Finalize Resolutions**

In his keynote, U.S. Ambassador Kolker illuminated that it was out of character for a number of countries in the AMRO region to vote in favor of LGBTQ recognition and advised the delegates to be careful to accurately represent their country's official positions in this simulation of international debate.

Similar to the resolutions of most regions, amendments proposed by countries of other regions during plenary were friendly and passed with little contention. The most substantive changes made included an amendment that removed a line requesting NGO collaboration in the WHO audit, which was recommended prior to implementing a universal health coverage system and an amendment to focus on evidence-based outcomes to determine universal health coverage success. Finally, a clause that provisioned for data protection throughout universal health coverage was added.

## SURVEY FINDINGS AND DISCUSSION

In total, 124 and 113 attendees from around the world registered for the AMWHO 2014 and 2015 conferences, respectively. At the conclusion of each conference, we conducted an anonymous online survey of delegates to seek feedback on their experience, with 50 responses (40.3% response rate) following the 2014 conference and 39 responses (34.5% response rate) following the 2015 conference. Attendee feedback after each conference is used to inform successes and opportunities for growth in moving this nascent organization forward.

### Successes

The vast majority (98%) of respondents from the AMWHO 2014 conference indicated the conference as being "good" or "better", and 90% of respondents indicated they would recommend the conference to a friend. Similarly, the majority (90%) of respondents from the AMWHO 2015 conference rated the experience as "good" or "better", and 97% indicated they would recommend AMWHO to a friend.

The vast majority of conference survey respondents rated the conference "good" or "better" and said they would recommend it to a friend.

Survey respondents were also asked to summarize their AMWHO experiences. Table 5 presents some of the open-ended feedback received from respondents according to the objectives that AMWHO seeks to realize. For example, respondents indicated AMWHO provided opportunities to gain practical experience in formulating global health policy that is not possible in a classroom environment. Other respondents pointed to AMWHO's ability to allow participants to think from the perspective of policy makers and other stakeholders.

**TABLE 5.** Feedback About the American Mock World Health Organization (AMWHO) Conferences: Qualitative Findings From Survey Respondents

AMWHO Objectives	Relevant Qualitative Survey Findings
Student engagement in global health diplomacy	Delegates noted that "AMWHO 2014 was a truly unique event; tackling international issues in healthcare by engaging in constructive debates and drafting resolutions allowed us to think critically about … solutions to promote health worldwide" and was "an ingenious way to engage students from various disciplines through *interactive and self-guided learning.*"
Guidance to future directions	One delegate reflected, "Coming with very little experience in the global public health arena, *AMWHO has allowed me to figure out where my purpose is*: at the intersection of public policy and public health. I truly give AMWHO significant credit for giving me clarity regarding my future plans." Another attendee said, "[AMWHO] *revived my passion for diplomacy and advocacy.* This was the first time I saw that my dream could actually be realized and that my potential and the possibilities are endless."
Putting theory and knowledge into practice	Several students commented on the practical nature of the conference. For example, 1 participant observed, "I learned that the intricacies and difficulties of forming global health policy are not something you can really learn in a lecture. The kinds of experiences provided at *AMWHO allowed a paradigm shift not possible in class*."
Understanding other perspectives	One student remarked, "AMWHO is a unique opportunity because *we learn to think in the perspectives of policymakers, which helps us understand the difficulties involved with it.* As future public health leaders, it's important to understand why nations or parties support or deny certain policies. *If we can understand someone's perspectives, only then can we begin to cooperate* with him/her to create more comprehensive solutions to our world's greatest problems."
Introducing diplomacy and global health skills	One student noted, "As a scientist, it is important for me to understand how health policy is made since it both influences and is influenced by scientific research. *Because of AMWHO I feel much better prepared to be an active participant in bridging the gap between research and global health*."

In addition, the International Federation of Medical Students' Associations (IFMSA) contact at WHO headquarters indicated that the AMWHO 2014 resolutions were succinct and practical and that only a few amendments exceeded the limitations of the WHO; as a whole, the IFMSA contact indicated the resolutions were similar in style, content, and urgency as those created in the WHA. AMWHO will receive feedback on the 2015 conference by March 2017.

### Obstacles

Two major challenges identified in the AMWHO 2014 conference were the plenary structure and the desire for professional development opportunities. The AMWHO 2015 conference provided an opportunity to grow and address these challenges to improve participants' experience.

#### Plenary

Many participants found the AMWHO 2014 plenary session to be less engaging and productive than the regional bloc sessions. In response, the dais of AMWHO 2015 received additional training to enable them to better facilitate debate, and we imposed time limits for the plenary debate of each resolution. We also placed limits on the number of resolutions each regional bloc could submit to plenary. The 2015 feedback showed that while plenary improved, there is still room for improvement in delegate engagement during plenary debate.

#### Professional Development Opportunities

Based on 2014 feedback, AMWHO 2015 expanded its professional development and networking opportunities. As a direct result, we hosted the "Lunch and Learn" event on Day 2, during which professionals from a variety of fields and sectors, e.g., business, education, and academia, were invited to eat with students and discuss their careers, paths, and interests. Another purpose of the "Lunch and Learn" event was to expose students to the various fields necessary to realize global health equity. Based on survey responses, the "Lunch and Learn" event was very popular among participants as a professional development tool. Additional networking opportunities included a "Delegate Social" held on Day 1.

## CONCLUSION

AMWHO fills a gap in global health policy education by providing students with exposure to the mechanics and political dynamics of the World Health Assembly, and an experiential opportunity to develop skills essential to careers in global health governance. After 3 international conferences, AMWHO has developed an effective structure, with post-conference survey results indicating that AMWHO fulfills its objectives.

The American Mock World Health Organization fills a gap in global health policy education by providing students with the opportunity to develop skills essential to careers in global health governance.

Since the creation of the conferences, AMWHO has sought to sustain its experiential education efforts by creating university chapters that engage in mock debate and that host regional conferences throughout the year (Table 3). This has filled an unmet need on campuses with growth from 5 to 12 chapters in 2016 alone.

AMWHO is working toward a future as a registered nonprofit that is in all high school and collegiate institutions in the United States, similar to Model United Nations. As AMWHO grows in scope, its purpose remains to develop, improve, and expand our capacity to create a generation of students who understand the complexities of diplomacy and policymaking. With continued growth, the organization has potential to contribute to developing a cadre of learned, well-trained, and capable global health practitioners for years to come.

## Notes

**Peer Reviewed**

## APPENDIX.

### Resolution of the Americas Region (AMRO) Sent to Plenary at the 2015 American Mock World Health Organization

Code: Resolution AMRO 2.1

Committee: AMRO

Subject: Ensuring universal health coverage as defined by equitable access to health services for all, with an emphasis on access to care for vulnerable populations

Sponsors: Costa Rica, Grenada, Haiti

Signatories: Argentina, Brazil, Canada, Chile, Colombia, Cuba, Dominican Republic, Ecuador, Honduras, Jamaica, Mexico, Peru, Saint Lucia, Uruguay

*Defining* vulnerable populations as sectors that currently lack or are at the risk of lacking access to health services,

*Recognizing* that vulnerable populations vary depending on the individual nation, including but not limited to: marginalized groups such as refugees, documented and undocumented immigrants, those of low socioeconomic status, mothers, children, the elderly, and LGBTQ+ populations, as well as geographically isolated populations or populations at high risk of natural disasters or violent conflict,

*Affirming* the need for the availability of a baseline set of health services for all people,

*Noting* that having a functional health information system is a prerequisite for achieving efficient and quality health care provision for all populations,

*Realizing* that the optimal method of health system financing varies among nations,

*Recognizing* that a successful UHC system must be sustainable,

#### The General Assembly Plenary,

1. *Requests* the creation of a health information system to collect data and monitor outcomes to tailor health systems to the needs of a given nation or area
  - *Requests* that individual nations assess health care needs and available resources by creating health information systems to collect and analyze data and monitor outcomes
    - *Recommends* that the World Health Organization (WHO) audit solutions that implement UHC as they're being carried out in these regions
    - *Recommends* that all systems will be maintained with the highest standard of data protection to ensure confidentiality and security
  - *Suggests* a focus on evidence-based outcomes in determining the success level of a UHC program
2. *Urges* the provision of a basic set of services to be available to all, prioritizing promotion of primary and preventative care, emphasizing maternal and child care
  - *Strongly suggests* that the basic scope of services in a given nation be expanded beyond the three broad areas stipulated above to encompass specific health needs of each country
    - *Strongly urges* that the issue of mental health be made a priority when deciding to expand the scope of basic services provided
    - *Recommends* results-based implementation of services in accord with the data generated by health information systems and encourage innovative determinations of success in areas (such as mental health) for which data-base metrics of success do not completely address the situation
3. *Endorses* the development by member states of systems for Universal Health Care (UHC) in which all populations with a focus on aiding vulnerable ones, have access to needed health services without incurring financial hardships
  - *Encourages* the creation of partnerships and collaborations between countries, which may be necessary to make the development of such a system feasible in some developing nations
  - *Encourages* better targeting and coordination of existing funds to address international issues, including but not limited to migrant populations, disease epidemics, international information databases, and assistance to countries in need with regards to individual universal healthcare coverage systems.
  - *Supports* the exploration of various financing schemes for individual contributions on a country-by-country basis
  - *Affirms* countries' responsibility to develop cost-effective health coverage systems
    - *Emphasizes* strengthening prevention-based programs as a way to potentially lessen financial burdens from preventable diseases, especially among vulnerable populations
4. *Urges* the expansion of health services into underserved regions to provide primary and preventive health services
  - *Suggests* the development of health facilities in both rural and urban regions to help lessen the geographical barriers and opportunity cost to health care access for vulnerable populations
  - *Encourages* the development of an able and plentiful health workforce to ensure that people of all regions have access to quality health care
    - *Suggests* that the aforementioned workforce also serve to educate the local populous with regards to personal healthcare
    - *Calls* upon both individual nation as well as the international community to begin a self-sustainable training program, focusing on developing community health worker programs
    - *Calls* to be accomplished via the building of human capital of local individuals from communities and local indigenous groups. This will ensure sustainability, cultural relevancy, and trust from locals of health workers.
      1. *Encourages* the formation of Community Health Workers (CHW) programs that are integrated with existing or developing health systems, including supervision and remuneration
  - *Suggests* utilizing existing and local infrastructure as well as creating and improving new health care infrastructure to maximize efficient resource allocation and ensure cultural sensitivity
5. *Expresses* its appreciation for Non-Governmental Organizations (NGO) participation, and encourages focused exit strategies for countries to remain self-reliant and sustainable
